# The Moderating Effects of Technostress Inhibitors on Techno-Stressors and Employee's Well-Being

**DOI:** 10.3389/fpsyg.2021.821446

**Published:** 2022-01-10

**Authors:** Yong Hang, Ghulam Hussain, Anam Amin, Muhammad Ibrahim Abdullah

**Affiliations:** ^1^College of Economics and Management, Jiangsu Maritime Institute, Nanjing, China; ^2^Department of Management Sciences, COMSATS University Islamabad, Lahore, Pakistan; ^3^Department of Management Sciences, COMSATS University Islamabad, Sahiwal, Pakistan

**Keywords:** health psychology, employees performance, occupational health psychology, techno-stressors, technostress inhibitors, well-being

## Abstract

This study determined the effects of techno-stressors on employees' well-being. It also determined the moderating role of technostress inhibitors in techno-stressors and employees' well-being. We employed a time-lagged design and self-administered survey method to collect data from banking employees. We retrieved 355 usable responses. The results showed that techno-stressors significantly and negatively affected employees' well-being. Technostress inhibitors significantly and positively affected the employee's well-being. The moderating effects of techno-stressors and technostress inhibitors showed that six of nine moderating effects were significant and positive. The results implied that technostress inhibitors help to improve employees' well-being. In the end, we present some implications for theory and practice.

## Introduction

Too much interdependence on technology to perform a job has side effects, especially for the employee's health and well-being. The stress arising from the use of technology is called technostress (Ragu-Nathan et al., 2008; Ayyagari et al., 2011). Technostress refers to stress that individuals experience because of information systems at the workplace (Tarafdar et al., 2019). It is a syndrome of adaptation because of the incompetence in managing advanced technologies (Brod, 1984). Individuals feel anxiety and the opposing effects on beliefs, deeds, insolences, and bodies when dealing with technology (Kupersmith, 1992; Weil and Rosen, 1997). Technostress results from distorted behaviors of work that are because of the use of updated information technologies at work and home (Srivastava et al., 2015).

People experience technostress when they cannot adjust to information technologies properly. Technology has become a part of our daily life, and it keeps us linked all the time (Tarafdar et al., 2014). People feel bound to share daily updates, answer work-related information on time and involve in routine multi-tasking. Researchers have specified the negative aspects of technological advancement (Heinssen et al., 1987; Fisher and Wesolkowski, 1999; Ma et al., 2021). Employees continuously update their technical skills and receive training to work with a new and upgraded technology-based system (Richardson, 2017). These requirements negatively affect employees' thoughts on the road to technological advancements (Heinssen et al., 1987). This situation forces employees to work hard and faster to fulfill work demands. Introducing new technology or system up-gradation may lead to downsizing. Employees feel vulnerable that they may be replaced if they cannot meet their job demands (Bradley, 2000).

Different researchers have studied the effects of technostress on a variety of outcomes, such as productivity, role overload and role conflict (Tarafdar et al., 2007), satisfaction and commitment (Ragu-Nathan et al., 2008; Ayyagari et al., 2011), disruptive behavior dissatisfaction at work, lack of job involvement, and poor job performance (Tarafdar et al., 2011, 2014; Srivastava et al., 2015), emotional exhaustion and poor work-life balance (Ma et al., 2021) and job insecurity (Grant et al., 2013). Earlier studies had mainly considered techno-stressors a composite construct that hindered the ability to segregate the effects of different techno-stressors on outcomes. Besides, their focus remained on work-related outcomes. However, non-work-life outcomes received less attention (Tarafdar et al., 2019). The researchers suggested that the social consequences of technology are not fully explored (Tarafdar et al., 2019; Zhong, 2020; Dai et al., 2021; Ma et al., 2021). Atasanoff and Venable (2013) called for an inquiry to understand the influence of technostress on employees' wellness. Employee's well-being as an important employee-level outcome has not been studied as a social consequence of technology that leaves a notable gap in the literature. To fill this void, this study mainly establishes the relationship between techno-stressors and employees' well-being separately to deepen the understanding.

When organizations implement a new technological-based system or upgrade the existing systems, they introduce interventions to help employees work on a new system (Ragu-Nathan et al., 2008). The organizational efforts address two important aspects: technical and psychological. The technical aspect covers the technical competencies that enable employees to work efficiently on a new system. The psychological aspect prepares employees to accept new technology, and it also covers the social support provided to an employee to work on a new system. These planned interventions are techno-stress inhibitors that help employees develop technical competencies to cope with stress stemming from technology (Ragu-Nathan et al., 2008). Thus, this study also explains the moderating role of technostress inhibitors in techno-stressors and employees' well-being. We conducted this study in the banking sector of Pakistan. Earlier studies could not generalize to Pakistani organizations because of differences in technology level and workforce characteristics. The banking sector of Pakistan is suitable to study the phenomena under investigation. This sector has undergone major reforms by introducing the new technological-based banking system.

The rest of this manuscript comprises four sections. The second section offers the hypotheses. The third section discusses the research method employed to test the hypotheses. The fourth section presents the findings of the study. The last section discusses the study's results and implications for theory and practice. The manuscript ends with some suggestions for future inquiry.

## Theory and Hypotheses

### Job Demands-Resources Model

This study uses the theoretical lens of the job demands-resources model (Bakker et al., 2003). This model posits that occupational stress results from the imbalances between the individual's job demands and the resources he/she is provided to meet job demands. Job demands refer to “*physical, social, or organizational characteristics of a job that require sustained physical or mental effort and therefore associated with psychological and physiological costs*” (Bakker et al. 2005, p. 170). Within the parlance of the job demands-resources model, the techno-stressors are the job demands that are the characteristics of jobs in the modern workplace. The job resources are “*physical, psychological, social or organizational aspects of the job that (a) are functional in achieving work goals, (b) reduce job demands and the associated physiological and psychological costs, or (c) stimulate personal growth and development*” (Bakker et al. 2005, p. 170). Technostress inhibitors such as literacy facilitation, technical support provision, and involvement facilitation are the organizational mechanisms expected to reduce techno-stressors' negative effects on employees' well-being.

### Techno-Stressors

Techno-stressors refer to the causes that create stress in an organization's environment because of information and communication technologies (ICTs) (Tarafdar et al., 2010). ICTs create stress in different ways (Ayyagari et al., 2011). Earlier researchers presented five technology-related factors that cause technostress; techno-overload, techno-invasion, techno-uncertainty, techno-complexity, and techno-insecurity (Ragu-Nathan et al., 2008; Tarafdar et al., 2010). *Techno-overload* refers to the situations which compel employees to work faster and longer (Ragu-Nathan et al., 2008; Tarafdar et al., 2014). *Techno-invasion* creates a blurring of boundaries between work and personal life perspectives; because individuals feel continuously connected (Tarafdar et al., 2010). Because of technology, employees feel they can be reached and contacted anytime (Ragu-Nathan et al., 2008). *Techno-complexity* means employees cannot manage the complexity of the new technology. They feel a lack of computer skills that force them to spend more time learning and understanding new technology (Ragu-Nathan et al., 2008; Tarafdar et al., 2014). *Techno-insecurity* relates to the state in which employees fear being substituted by experienced people with greater ICT skills. Because of continuous changes in hardware and software, the situation becomes very uncertain for employees and refers to *techno-uncertainty*. Employees feel organizations move from one cycle to another with reduced time between systems upgrades, leaving them unsettled (Ragu-Nathan et al., 2008).

### Technostress Inhibitors

An organization's survival depends on its ability to adopt new technology. Technology implementation is not simple as it changes the social performance, distresses persons and groups in the organization (Nelson, 1990). An organization invests resources in helping its employees to cope with the technical and social changes arising because of introducing new technology. Technostress inhibitors are such organizational resources/mechanisms that reduce the technostress among employees (Ragu-Nathan et al., 2008). Technostress inhibitors comprise literacy facilitation, technical support provision, and involvement facilitation. *Literacy facilitation* is defined as a mechanism to share ICT-related knowledge within an organization through professional training or documentation (Ragu-Nathan et al., 2008; Shah et al., 2021). *Technical support provision* refers to technical support provided to users to solve technology-related problems (Ragu-Nathan et al., 2008; Tarafdar et al., 2011). *Involvement facilitation* is defined as keeping the individual informed about the rationale of introducing new technology, involving them in system introduction to progress and application (Ragu-Nathan et al., 2008; Tarafdar et al., 2011).

### Employees Well-Being

Well-being is a subjective concept and refers to an individual's overall evaluation of his/her quality of life depending on his/her standards and emotional experience (Diener, 2000; de Jong, 2014). It is a state of individual being characterized by good health or wellness and comfort, home life, and personal prosperity (Seligman, 2011). It refers to the overall excellence of an employee's familiarity and functioning at work. It creates an environment that encourages a state of satisfaction and permits an employee to flourish and achieve his/her full potential to benefit himself/herself and the organization (Grant et al., 2007; Sarfraz et al., 2020). Well-being encompasses two aspects; subjective experience of happiness and realization of personal achievement and self-actualization (Bayhan Karapinar et al., 2020). Changes in the individuals' situations or circumstances, including their work context, affect their well-being. Employers are increasingly adopting measures that promote the health and well-being of their employees. Organizations have accepted that the work environment can encourage or strengthen good working practices and routine varieties positively contribute to employees' physical and psychological well-being. For example, an effort to promote job quality that permits employees more control, independence, and participation positively contributes to employees' well-being (Coats and Lekhi, 2008; Ajaz et al., 2020).

### Techno-Stressors and Employee's Well-Being

In the fast-changing environment, organizations continuously upgrade their work-related technology for survival. Though introducing new technology enhances work efficiency, it also negatively affects employees. Because of the new technological system, employees experience system crashes, face data transfer errors, and poor technical support that cause frustration and create stress. Further, organizational efforts to urge employees to fulfill tasks rapidly and work for a longer time cause frustration among employees (Ragu-Nathan et al., 2008). This implies that techno-stressors negatively affect employees' well-being. For example, because of techno-overload, employees work for long hours and faster to fulfill their job demands (Fisher and Wesolkowski, 1999). Techno-invasion is when technology forces employees to deal with work issues even when they are at home and keeps them attached to their work and organization. Techno-complexity requires employees to invest more time and effort to learn new technological work procedures at work or during their time (Ragu-Nathan et al., 2008). Investing extra time and effort drains employees' resources reservoir that negatively affects their well-being (Ma et al., 2021). Techno-insecurity and techno-uncertainty promote feelings of fear and insecurity among employees.

The fear of job loss reduces employees' confidence to work with new technology, leading to anxiety and frustration (Tarafdar et al., 2011). Techno-stressors are the job demands related to the use of technology that individuals find unable to meet and leaving adverse effects on their well-being (Tarafdar et al., 2019). Employees unable to meet job demands during normal working hours have to work for late hours to fulfill their job demands. Work-related activities beyond the normal working hours take time away from their leisure and relaxing activities and shorten time to recover from work-related stress (Atasanoff and Venable, 2013). Besides, the job demands-resources model and the effort-recovery model (Meijman and Mulder, 1998) provide theoretical explanations of the negative relationships between techno-stressors and well-being. This model also states that employees facing tough work conditions spend excessive time and energy at work, leaving little time to engage in activities that help them recover from stress work-related stress (Deng and Gao, 2017; Bayhan Karapinar et al., 2020; Abdullah et al., 2021). Therefore, we expect that techno-stressors negatively affect employees' well-being.

H1: Techno-stressors (techno-overload, techno-invasion, techno-complexity, techno-insecurity, and techno-uncertainty) have significant and negative effects on employees' well-being.

### Technostress Inhibitors and Employee's Well-Being

The technostress inhibitors are organizational resources that are expected to play dual roles; (1) they are positively associated with employees' well-being, (2) they work as boundary conditions to buffer the negative effects of techno-stressors on employee's well-being (Ragu-Nathan et al., 2008).

When new ICTs are implemented, organizations train their employees to build and improve their competencies to work efficiently on a new system (Yaverbaum, 1988; Zorn, 2002). The managers construct some model demonstration and make plans for practice (Kupersmith, 1992). During the application of complex systems, systematic workload reduction sometimes gives to employees to study and practice on a new system (Brod, 1984). Similarly, the users' involvement in system planning and implementation phases is another mechanism for reducing the stress (Brod, 1984). By welcoming the individuals to join the discussion on how new applications can be executed and integrating their requirements into system design increases their confidence in the new system. Helping employees to become familiar with new applications reduces the stress among them and increases their well-being (Nelson, 1990). Earlier research also showed that technostress inhibitors have a positive role and lessen stressors' effects (Ragu-Nathan et al., 2008; Ma et al., 2021). Thus, we hypothesize that technostress inhibitors positively relate to employees' well-being.

H2: Technostress inhibitors (literacy facilitation, technical support provision, and involvement facilitation) have significant and positive effects on employees' well-being.

### The Moderating Effects of Technostress Inhibitors on Techno-Stressors and Employee's Well-Being

**Figure 1:**
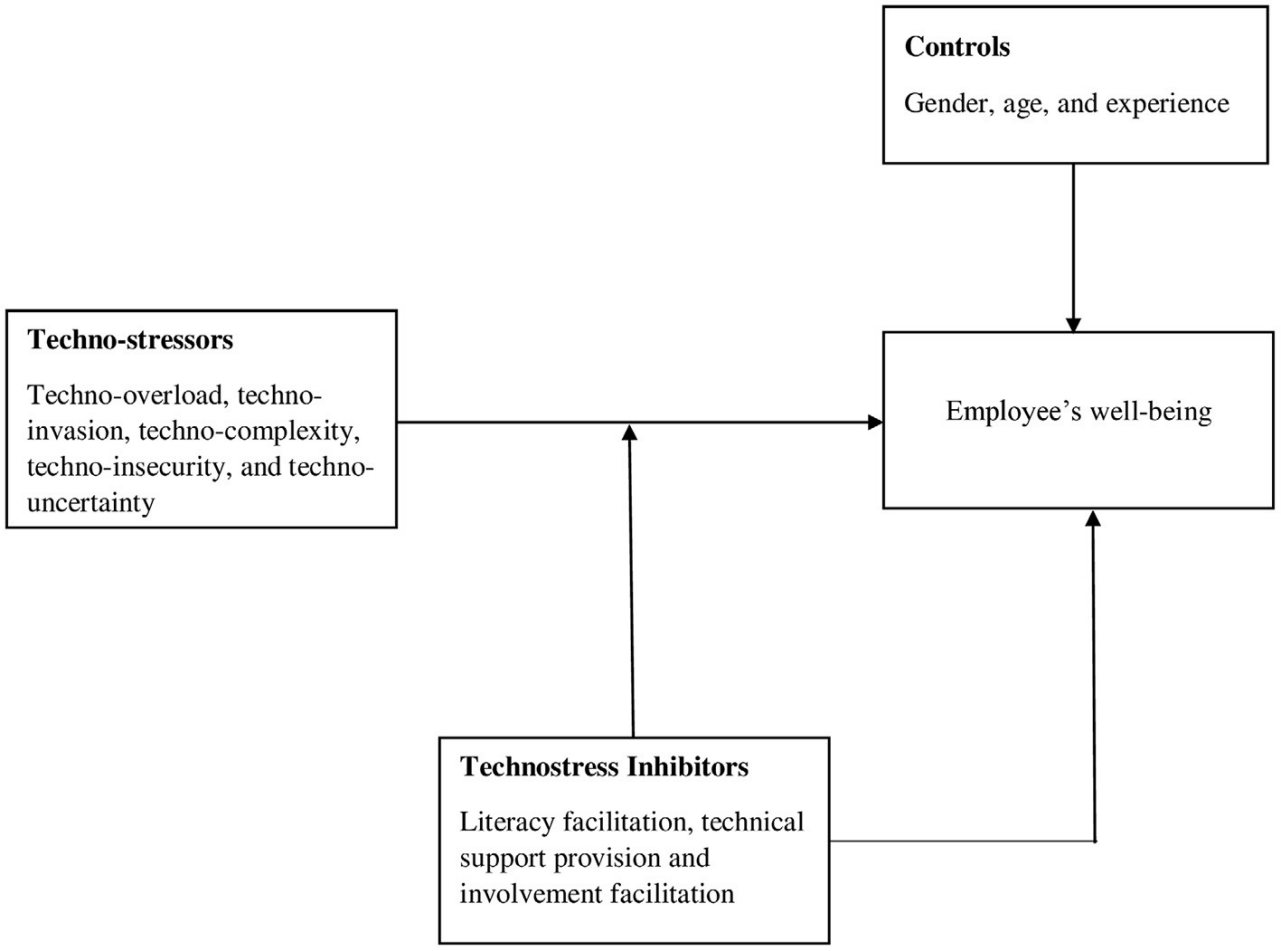
The research framework.

As already discussed, technostress inhibitors are the organizational resources that reduce or inhibit techno-stressors' outcomes (Ma et al., 2021). These technostress inhibitors work as boundary conditions to reduce the effects of techno-stressors on employees' well-being. They encompass mechanisms like providing system-related technical training to employees, helping employees to troubleshoot the problems, and involving them in designing and implementing systems that can ensure the successful implementation and execution of the new system. These are confidence-building measures that increase employees' confidence in new systems and organizations. These make it easier to use and lead to positive job appraisals among individuals. Therefore, on one side, they positively contribute toward employee-level outcomes. They also offer that much potential that can neutralize the negative effects of techno-stressors on employee-related outcomes (Ragu-Nathan et al., 2008). Earlier research has used the composite scores of techno-stressors and technostress inhibitors to determine their joint effects on outcomes. This approach hinders the segregation of the effects. Thus, to deepen the understanding, this study will determine the dimensional joint effects of techno-stressors and technostress inhibitors on employees' well-being. Thus, we hypothesize that;

H3: Technostress inhibitors (literacy facilitation, technical support provision, and involvement facilitation) moderate the relationship between techno-stressors (techno-overload, techno-invasion, techno-complexity, techno-insecurity, and techno-uncertainty) and employee's well-being, such that their join effects will be positive on employee's well-being when technostress inhibitors are high and vice versa (Figure 1).

## Research Methodology

### Population and Sample

The researchers targeted a large private bank operating in Pakistan, which has recently implemented a new technological system in its banking operations. Because of resource constraints, we restricted our sample to one administrative region of the bank. We got a list of branches employees that contained 1990 employees. We treated 1990 as our target population and employed a probability sampling method; we contacted every 3rd subject for data collection. We approached the respondents with the consent of management.

### Data Collection Method and Procedure

We used a time-lagged design and self-administered survey method to collect the data from the respondents. We prepared two booklets of the questionnaires. The first booklet comprises demographics and scales of techno-stressors and technostress inhibitors. The second booklet comprises the scale of well-being. In preparing the first booklet, we followed the guidelines of Brannick et al. (2010). To create the psychological separation, the booklet further comprises three sections. The first section comprises the demographics of the respondents. The second section comprises the measurement scale of techno-stressors. The third section comprises the measurement scale of technostress inhibitors. We also included the definitions of the variables in each section. Each survey accompanied a cover letter that explained the study's aim and the anonymity of the responses. We did not involve any third person in distributing and collecting the filled surveys to ensure the confidentiality of the responses.

We assigned a unique code to each respondent to match their responses of first (T1) and second (T2) waves. In the first wave (T1), we distributed over 650 questionnaires to the respondents and retrieved 450 filled questionnaires. Some questionnaires were incomplete or inappropriately filled. After discarding such responses, we had 402 valid responses at the end of the first wave. We maintained a lag of three months and distributed 402 copies of the second booklets in the second wave to those respondents who had valid responses at the end of the first wave and retrieved 355 filled responses at the end of the second wave. The demographic profile of the respondents showed there were 265 male respondents and 90 female respondents. Further, 62 respondents were bachelor's degree holders and 293 master's degree holders.

### Instruments

#### Techno-Stressors

We used 21 items of Ragu-Nathan et al. (2008) to measure techno-stressors' five dimensions: techno-overload, techno-invasion, techno-uncertainty, techno-complexity, and techno-insecurity. Of these 21 items, five items for techno-overload and four items each for techno-invasion, techno-uncertainty, techno-complexity, and techno-insecurity were used.

#### Technostress Inhibitors

Technostress inhibitors comprise three factors: literacy facilitation, technical support provision, and involvement facilitation. The eleven-item scale of Ragu-Nathan et al. (2008) was used to measure technostress inhibitors. Of these 11 items, four items were used for literacy facilitation and involvement facilitation and three for technical support provision.

#### Employees' Well-Being

The employee's well-being was measured using the eight-item scale of Diener et al. (2010). All items were scored on a five-point Likert scale ranging from strongly disagree “1” and strongly agree “5.”

#### Control Variables

We controlled for the effects of gender, age, and experience because of their potential heterogeneity effects on employees' well-being.

## Results

This section presents the results of the study. First, we conducted a confirmatory factor analysis to test the validity and reliability of the scales. Second, we reported the results of Harman's single factor test to test the possibility of common method variance. Third, we obtained the Pearson correlation coefficients to determine the degree and direction of association among the study's variables. Fourth, we tested the study's hypotheses through moderated multiple regression techniques. Table 1 shows measurement model results.

**Table 1:** Measurement model results.

**Construct**	**Indicators**	**Factor loading**	**AVE**	**Composite reliability**	**Cronbach's alpha**
Techno-overload	Techno-overload 1	0.869			
	Techno-overload 2	0.895			
	Techno-overload 3	0.872	0.758	0.94	0.92
	Techno-overload 4	0.879			
	Techno-overload 5	0.838			
Techno-invasion	Techno-invasion 1	0.861			
	Techno-invasion 2	0.858			
	Techno-invasion 3	0.894	0.744	0.921	0.885
	Techno-invasion 4	0.837			
Techno complexity	Techno-complexity 1	0.851			
	Techno-complexity 2	0.848			
	Techno-complexity 3	0.864	0.720	0.911	0.87
	Techno-complexity 4	0.831			
Techno-insecurity	Techno-insecurity 1	0.88			
	Techno-insecurity 2	0.888			
	Techno-insecurity 3	0.898	0.767	0.929	0.898
	Techno-insecurity 4	0.836			
Techno-uncertainty	Techno-uncertainty 1	0.823			
	Techno-uncertainty 2	0.866			
	Techno-uncertainty 3	0.886	0.742	0.92	0.884
	Techno-uncertainty 4	0.868			
Literacy facilitation	Literacy facilitation 1	0.873			
	Literacy facilitation 2	0.88			
	Literacy facilitation 3	0.884	0.748	0.922	0.887
	Literacy facilitation 4	0.822			
Technical support provision	Technical support provision 1	0.909			
	Technical support provision 2	0.887	0.796	0.921	0.872
	Technical support provision 3	0.881			
Involvement facilitation	Involvement facilitation 1	0.838			
	Involvement facilitation 2	0.804			
	Involvement facilitation 3	0.883	0.678	0.894	0.841
	Involvement facilitation 4	0.764			
Well-being	Well-being 1	0.743			
	Well-being 2	0.828			
	Well-being 3	0.688			
	Well-being 4	0.862			
	Well-being 5	0.81	0.629	0.931	0.914
	Well-being 6	0.818			
	Well-being 7	0.876			
	Well-being 8	0.693			

### Validity and Reliability

We tested the validity and reliability of the measurement scales through confirmatory factor analysis in Smart-PLS. We specified the indicators on their respective latent constructs and computed the algorithm. The results showed that loading scores in all cases exceeded the cut-off value, i.e., 0.50 (Hair et al., 2019). The average variance extracted (AVE) scores were >0.50, showing that measurement scales had good convergent validity. We tested discriminant validity by comparing the squared roots of AVE scores with paired correlation coefficients (Fornell and Larcker, 1981). The comparison revealed that squared roots of AVE scores were greater than paired correlation coefficients that confirmed the discriminant validity.

As for reliability, we obtained composite reliability and Cronbach's Alpha scores. The values of reliability measures were >0.70 that showed measures employed in this study had good reliability (Hair et al., 2019). After revealing the good reliability and validity, we computed the average itemized scores of the latent constructs.

### Common Method Variance

Despite employing time-lagged design and some procedural remedies, data collected through a single source could be subject to common method bias. We used Harman single factor test through exploratory factor analysis (EFA) to determine the possibility of common method bias in our data. The results showed that the first factor accounted for only 24% of the total variance, <50%, showing that common method variance is not a significant threat to our study's results (Podsakoff et al., 2003). Further, EFA with varimax rotation produced the correct number (nine) of factors that accounted for 75% of the total variance. These results show that common method variance effects are nonsignificant in our data.

### Correlation

We computed the bivariate Pearson correlation coefficient to determine the degree and direction of association among the study's variables. The correlation values with a single asterisk (^*^) are significant at *p* < *0.05*, and correlation values with double asterisks (^**^) are significant at *p* < *0.01*. The correlation values without asterisks are non-significant. The significant correlation values indicate that the regression model would produce significant effects at the time of the test of hypotheses. The descriptive statistics such as mean and standard deviation of the latent constructs are reported in Table 2.

**Table 2:** Descriptive statistics, correlation, and squared roots of AVE scores.

	**Mean**	**SD**	**1**	**2**	**3**	**4**	**5**	**6**	**7**	**8**	**9**	**10**	**11**	**12**
1. Gender	-	-												
2. Age	32.13	8.04	−0.261^**^											
3. Experience	5.54	2.70	−0.277^**^	0.872^**^										
4. Techno-overload	2.051	0.958	0.037	−0.014	−0.018	**(0.871)**								
5. Techno-invasion	2.265	0.859	−0.001	−0.060	−0.040	0.291^**^	**(0.863)**							
6. Techno-complexity	2.391	0.942	−0.036	−0.010	0.022	0.174^**^	0.165^**^	**(0.849)**						
7. Techno-insecurity	2.088	0.961	−0.070	0.035	−0.005	0.066	0.025	−0.028	**(0.876)**					
8. Techno-uncertainty	2.264	1.023	−0.071	−0.086	−0.075	0.109^*^	0.066	−0.105^*^	−0.156^**^	**(0.861)**				
9. Literacy facilitation	3.791	0.854	0.059	−0.037	−0.044	−0.189^**^	−0.198^**^	−0.110^*^	−0.183^**^	−0.204^**^	**(0.865)**			
10. Technical support provision	3.802	0.879	0.009	0.038	0.036	−0.225^**^	−0.449^**^	−0.592^**^	−0.001	0.016	0.171^**^	**(0.892)**		
11. Involvement facilitation	3.903	0.814	0.030	−0.021	−0.011	−0.164^**^	−0.135^*^	−0.144^**^	−0.672^**^	0.034	0.232^**^	0.131^*^	**(0.823)**	
12. Well-being	4.051	0.608	0.112^*^	−0.168^**^	−0.114^*^	−0.411^**^	−0.322^**^	−0.304^**^	−0.292^**^	−0.115^*^	0.369^**^	0.416^**^	0.319^**^	**(0.793)**

* *p < 0.05*,

** *p < 0.01*.

*Bold values in parenthesis are the squared roots of AVE scores, 1-gender, 2-age, 3-experience, 4-techno-overload, 5-techno-invasion, 6-techno-complexity, 7-techno-insecurity, 8-techno-uncertainty, 9-literacy facilitation, 10-technical support provision, 11-well-being*.

### Hypotheses Testing

We tested the hypotheses using the multiple moderated regression technique in SPSS (see Table 3). This technique allows defining the hierarchy and computing the variance accounted for each set of predictors. Before running the moderated multiple regression analysis, we computed the interaction terms of predictors (techno-stressors) and moderators (technostress inhibitors) using their standardized scores. The interaction term based on standardized scores of the predictor and moderator minimizes the possibility of multicollinearity (Aiken and West, 1991). We run a four-step hierarchical procedure to test the hypotheses. First, we entered the control variables in the model. Second, we entered the predictors (techno-stressors). Third, we entered the technostress inhibitors. Fourth, we entered the model's interaction terms of the predictors and moderators.

**Table 3:** Hypotheses testing.

		**Well-being**			
		**B**	**S.E**.	**t value**	**Δ*R*^2^**
Step 1 (Controls)	**Controls**				
	Constant	4.154	0.090	46.369^**^	0.039**
	Gender	0.113	0.076	1.485	
	Age	−0.208	0.079	−2.622^**^	
	Experience	0.132	0.093	1.422	
Step 2 (Predictors)	**Techno-stressors**				
	Techno-overload	−0.169	0.027	−6.240^**^	0.356^**^
	Techno-invasion	−0.118	0.027	−4.400^**^	
	Techno-complexity	−0.153	0.026	−5.817^**^	
	Techno-insecurity	−0.174	0.026	−6.657^**^	
	Techno-uncertainty	−0.095	0.026	−3.571^**^	
Step 3 (Moderators)	**Technostress inhibitors**				
	Literacy facilitation	0.106	0.026	4.015^**^	0.056^**^
	Technical support provision	0.138	0.034	4.094^**^	
	Involvement facilitation	0.012	0.034	0.358	
Step 4 (Interaction terms)	**Interaction terms**				
	Techno-overload × literacy facilitation	0.092	0.042	2.207^*^	0.139^**^
	Techno-overload × technical support provision	0.134	0.039	3.436^**^	
	Techno-overload × involvement facilitation	−0.016	0.037	−0.436	
	Techno-invasion × literacy facilitation	0.105	0.035	3.004^**^	
	Techno-invasion × technical support provision	−0.028	0.027	−1.033	
	Techno-invasion × involvement facilitation	−0.068	0.033	−2.037^*^	
	Techno-complexity × literacy facilitation	0.089	0.036	2.438^*^	
	Techno-complexity × technical support provision	0.079	0.028	2.832^**^	
	Techno-complexity × involvement facilitation	−0.022	0.030	−0.726	
	Techno-insecurity × literacy facilitation	0.062	0.027	2.296^*^	
	Techno-insecurity × technical support provision	−0.100	0.035	−2.857^**^	
	Techno-insecurity × involvement facilitation	−0.056	0.025	−2.240^*^	
	Techno-uncertainty × literacy facilitation	−0.012	0.025	−0.473	
	Techno-uncertainty × technical support provision	−0.009	0.032	−0.290	
	Techno-uncertainty × involvement facilitation	0.010	0.038	0.266	

* *p < 0.05*,

** *p < 0.01. Bold values in the column are proportion variance explained by predictors at each step*.

The results of a four-step moderated multiple regression model are shown in Table 3. The first step results showed that gender (β = 0.113, ns) and experience (β = 0.132, ns) did not significantly influence the employee's well-being. Respondents' age significantly but negatively affected employees' well-being (β = −0.208, *p* < 0.01). The control variables accounted for 3.9% of the employees' being variance.

The results of the second step showed that all five predictors; techno-overload (β = −0.169, *p* < 0.01), techno-invasion (β = −0.118, *p* < 0.01), techno-complexity (β = −0.153, *p* < 0.01), techno-insecurity (β = −0.174, *p* < 0.01), and techno-uncertainty (β = −0.095, *p* < 0.01) significantly and negatively influenced the employee's well-being. The techno-stressors accounted for 35.6% of the total employee variance. The results of the third step showed that two of the three technostress inhibitors produced significant and positive effects on employees' well-being. Literacy facilitation (β = 0.106, *p* < 0.01) and technical support provision (β = 0.138, *p* < 0.01) significantly and positively affected the employee's well-being. However, involvement facilitation did not show a significant effect (β = 0.012, ns) on employees' well-being. Technostress inhibitors explained 5.6% of the variance in employees' well-being.

The results of the fourth step showed that out of fifteen possible moderating effects, nine moderating effects are significant. Further, out of nine significant moderating effects, six are positive, and three are negative, contrary to the expectations. Literacy facilitation (β = 0.092, *p* < 0.05) and technical support provision (β = 0.134, *p* < 0.01) combined with techno-overload significantly and positively affected the employee's well-being. Techno-invasion joined with literacy facilitation (β = 0.105, *p* < 0.01) significantly and positively affected the employee's well-being. Techno-invasion combined with involvement facilitation significantly but negatively (β = −0.068, *p* < 0.05) affected the employee's well-being. Techno-complexity combined with literacy facilitation (β = 0.089, *p* < 0.05) and technical support (β = 0.079, *p* < 0.05) significantly and positively affected the employee's well-being. Techno-insecurity and literacy facilitation conjointly positively affected the employee's well-being (β = 0.062, *p* < 0.01). Techno-insecurity combined with technical support provision (β = −0.10, *p* < 0.01), and involvement facilitation (β = −0.056, *p* < 0.05) significantly but negatively affected the employee's well-being. The interaction terms accounted for 13.9% of the employees' well-being variance.

## Discussion

This section presents the outcomes of the study's findings in terms of theoretical and practical implications. We formulated the direct and moderating effects hypotheses on the premise of the job demands-resources model (Bakker et al., 2003, 2005). The first set of direct hypotheses was about techno-stressors' effects on employees' well-being. The techno-stressors are the job demands arising from upgrading or implementing a new technology system. The results showed that all five techno-stressors, such as techno-overload, techno-invasion, techno-complexity, techno-insecurity, and techno-uncertainty, negatively affected employees' well-being. Our study's results are consistent with the job demands-resources model (Bakker et al., 2005) and results of previous studies (Ragu-Nathan et al., 2008: Tarafdar et al., 2011; Ma et al., 2021). These results imply employees suffer most from technological changes in the organizations.

The second set of hypotheses was regarding the direct effects of technostress inhibitors on employees' well-being. The technostress inhibitors are the resources provided by an organization to its employees to cope and work with new technological systems. The results showed that technostress inhibitors such as literacy facilitation and technical support provision have significantly and positively affected the employee's well-being. The results are fully in line with the theoretical lens of the job demands-resources model (Bakker et al., 2005) and findings of the earlier studies (Ragu-Nathan et al., 2008: Tarafdar et al., 2011; Ma et al., 2021). The results imply that technostress inhibitors, as organizational resources, positively contribute toward employees' well-being.

In testing the third set of hypotheses regarding the moderating effects of technostress inhibitors on techno-stressors and employees' well-being, we tested fifteen possible interaction effects based on individual dimensions of techno-stressors and technostress inhibitors. The results showed that of the fifteen possible moderating effects, we found nine significant moderating effects. Of these significant effects, six interaction terms were positive and supported the hypothesized relationships. Among three technostress inhibitors, literacy facilitation combined with techno-overload, techno-invasion, techno-complexity, and techno insecurity positively affected employees' well-being. The results imply that building and improving employee competence through training programs is the most effective mechanism to build employees' confidence in new technology (Ragu-Nathan et al., 2008; Tarafdar et al., 2014), resulting in improved well-being. In combination with techno-overload and techno-complexity, technical support provision positively affected the employee's well-being. The results imply technical support provides assurance to employees to solve their system-related problems and increases their comfort level with a new system (Tarafdar et al., 2014). In combination with techno-invasion and techno-insecurity, involvement facilitation negatively affected the employee's well-being. The results are complex, but these may hold in a power distance society like Pakistan. The possible explanation of the negative moderating effect of involvement facilitation on techno-invasion and employees' well-being could be that employees might have felt over-involved, leading to frustration and exhaustion. When a new system is implemented, employees already feel insecure because of a lack of competence. Employee involvement could increase their fear of insecurity because they might feel that their competence is judged for the new system. The same also holds for the negative joint effect of techno-insecurity and technical support provision.

## Conclusion

This study determined the effects of techno-stressors on employees' well-being. It also explained the role of technostress inhibitors to buffer the negative effects of techno-stressors on employees' well-being. The time-lagged data (3-month apart) were collected from banking employees. The results supported the negative effects of techno-stressors on employees' well-being. The results also supported technostress inhibitors' direct and moderating roles in employees' well-being, with few exceptions. The results showed that technology causes anxiety and fear of insecurity among employees, negatively affecting their well-being. Organizations can help employees cope with anxiety and fear and build their confidence and competence to work on a new system by providing resources. In the end, we offered implications of the findings regarding theory and practice. We concluded our study by suggesting some promising areas for future researchers.

### Study Implications

This study significantly contributes to theory and practice. Our study complements and extends the job demands-resources model (Bakker et al., 2003, 2005) and technostress literature (Ragu-Nathan et al., 2008; Tarafdar et al., 2011, 2014, 2019; Ma et al., 2021). We established direct and indirect effects hypotheses based on the premise of the job demands-resources model. Our study extended the job demands-resources model and technostress literature by explaining the effects of techno-stressors on employee well-being that earlier studies overlooked. Second, we included all five techno-stressors in our model and segregated their effects on well-being. We expect our study to strengthen the understanding and provide a detailed picture to explain which techno-stressor is crucial for employees' well-being. For example, the individual effects of techno-stressors showed that the magnitudes of techno-insecurity, techno-overload, and techno-complexity are high. Implementing new technology heightens feelings of insecurity, followed by information overload and system complexity.

Similarly, we also explained technostress inhibitors' direct effects on employees' well-being and moderating effects on techno-stressors and employees' well-being. Our direct effects results showed that literacy facilitation and technical support provision showed significant and positive effects that suggest that training and technical support are crucial for a new system's success (Ragu-Nathan et al., 2008; Ma et al., 2021). The moderating effects showed that literacy facilitation played a significant role in helping employees cope with technostress that increased the employee's well-being. These findings are important for managers to understand that they should arrange system-related training programs to improve employees' competence. After literacy facilitation, technical support provision neutralized the negative effects of techno-stressors on employees' well-being. The managers should ensure that technical support is available to employees when working on the new system.

## Limitations and Future Research Directions

Despite its value, our study is not without limitations. First, we selected a sample from one organization that could restrict the generalizability of our study. Every organization has unique technology requirements, systems, culture, and work procedures. The types and levels of technology vary from industry to industry. The sample should be taken from multiple organizations for better generalization of the results. Some countries have high technology infrastructure and technology acceptance and usage compared to others. We invite future researchers to conduct cross-cultural studies comparing high and low-tech countries. Second, we suggest extending our model by taking employees' well-being as a mediating mechanism of work-related behavioral outcomes, such as performance, organizational citizenship behaviors, and counter-productive work behaviors. Besides employees' well-being, future researchers should explore other mediating mechanisms to offer an alternative explanation. Third, our study included organizational resources (technostress inhibitors) as mechanisms to cope with technostress. Besides organizational resources, personal-level and peer-level resources should be explored to buffer the negative effects of techno-stressors on outcomes under investigation. Fourth, involvement facilitation in our study did not provide the expected potential. Though, we understand that this could be because of sample homogeneity. We could also ascribe this to a national cultural context like in a power distance society like Pakistan, where end-users are just informed about the decisions. This could be one of the reasons that employees do not show disagreement with their managers and approve whatever top management has decided. We invite future researchers to conduct cross-cultural studies to understand the culture-specific nature.

## Data Availability Statement

The raw data supporting the conclusions of this article will be made available by the authors, without undue reservation.

## Ethics Statement

Ethical review and approval was not required for the study on human participants in accordance with the local legislation and institutional requirements. The patients/participants provided their written informed consent to participate in this study.

## Author Contributions

All authors listed have made a substantial, direct, and intellectual contribution to the work and approved it for publication.

## Conflict of Interest

The authors declare that the research was conducted in the absence of any commercial or financial relationships that could be construed as a potential conflict of interest.

## Publisher's Note

All claims expressed in this article are solely those of the authors and do not necessarily represent those of their affiliated organizations, or those of the publisher, the editors and the reviewers. Any product that may be evaluated in this article, or claim that may be made by its manufacturer, is not guaranteed or endorsed by the publisher.

## References

[B1] Abdullah M. I. Huang D. Sarfraz M. Ivascu L. Riaz A. (2021). Effects of internal service quality on nurses' job satisfaction, commitment and performance: Mediating role of employee well-being. Nurs. Open 8, 607–619. 10.1002/nop2.665 33570299 PMC7877139

[B2] Aiken L. S. West S. G. (1991). Multiple Regression: Testing and Interpreting Interactions. Thousand Oaks, CA: Sage Publications, Inc.

[B3] Ajaz A. Shenbei Z. Sarfraz M. (2020). Delineating the influence of boardroom gender diversity on corporate social responsibility, financial performance, and reputation. Logforum 16, 61–74. 10.17270/J.LOG.2019.376

[B4] Atasanoff L. Venable M. A. (2013). Technostress: implications for adults in the workplace. J. Career Dev. 65, 326–338. 10.1002/cdq.1211133084928

[B5] Ayyagari R. Grover V. Purvis R. (2011). Technostress: Technological antecedents and implications. MIS Q. 35, 831–858. 10.2307/41409963

[B6] Bakker A. B. Demerouti E. Euwema M. C. (2005). Job resources buffer the impact of job demands on burnout. J. Occup. Health Psychol. 10, 170–180. 10.1037/1076-8998.10.2.170 15826226

[B7] Bakker A. B. Demerouti E. Schaufeli W. B. (2003). Dual processes at work in a call centre: an application of the Job Demands-Resources model. Eur. J. Work. Organ. 12, 393–417. 10.1080/13594320344000165

[B8] Bayhan Karapinar P. Metin Camgoz S. Tayfur Ekmekci O. (2020). employee wellbeing, workaholism, work–family conflict and instrumental spousal support: a moderated mediation model. J. Happiness Stud. 21, 2451–2471. 10.1007/s10902-019-00191-x

[B9] Bradley G. (2000). The information and communication society: how people will live and work in the new millennium. Ergonomics 43, 844–857. 10.1080/001401300409053 10929821

[B10] Brannick M. T. Chan D. Conway J. M. Lance C. E. Spector P. E. (2010). What is method variance and how can we cope with it? a panel discussion. Organ. Res. Methods 13, 407–420. 10.1177/1094428109360993

[B11] Brod C. (1984). Technostress: The Human Cost of the Computer Revolution. Reading, MA: Addison-Wesley.

[B12] Coats D. Lekhi R. (2008). Good Work: Job Quality in a Changing Economy. London: Work Foundation.

[B13] Dai C. Tai Z. Ni S. (2021). Smartphone use and psychological well-being among college students in China: a qualitative assessment. Front. Psychol. 12:708970. 10.3389/fpsyg.2021.708970 34566786 PMC8458628

[B14] de Jong J. (2014). Externalization motives and temporary versus permanent employee psychological well-being: a multilevel analysis. Eur. J. Work Organ. Psychol. 23, 803–815. 10.1080/1359432X.2013.818217

[B15] Deng S. Gao J. (2017). The mediating roles of work–family conflict and facilitation in the relations between leisure experience and job/life satisfaction among employees in Shanghai banking industry. J. Happiness Stud. 18, 1641–1657. 10.1007/s10902-016-9771-8

[B16] Diener E. (2000). Subjective well-being: the science of happiness and a proposal for a national index. Am. Psychol. 55, 34–43. 10.1037/0003-066X.55.1.34 11392863

[B17] Diener E. Wirtz D. Tov W.. (2010). New well-being measures: short scales to assess flourishing and positive and negative feelings. Soc. Indic. Res. 97, 143–156. 10.1007/s11205-009-9493-y

[B18] Fisher W. Wesolkowski S. (1999). Tempering technostress. Technol. Soc. Mag. 18, 28–42. 10.1109/44.75224327295638

[B19] Fornell C. Larcker D. F. (1981). Evaluating structural equation models with unobservable variables and measurement error. J. Mark. Res. 18, 39–50. 10.1177/002224378101800104

[B20] Grant A. M. Christianson M. K. Price R. H. (2007). Happiness, health, or relationships? managerial practices and employee well-being tradeoffs. Acad. Manag. Perspect. 21, 51–63. 10.5465/amp.2007.26421238

[B21] Grant C. A. Wallace L. M. Spurgeon P. C. (2013). An exploration of the psychological factors affecting remote e-worker's job effectiveness, well-being, and work-life balance. Empl. Relat. 35, 527–546. 10.1108/ER-08-2012-0059

[B22] Hair J. F. Jr Black W. C. Babin B. J. Anderson R. E. Ttham R. L. (2019). Multivariate Data Analysis, 8th Edn. Noida: Cengage Learning EMEA, India.

[B23] Heinssen R. Glass C. Knight L. (1987). Assessing computer anxiety: development and validation of the computer anxiety rating scale. Comput. Hum. Behav. 3, 49–59. 10.1016/0747-5632(87)90010-0

[B24] Kupersmith J. (1992). Technostress and the reference librarian. Ref. Ser. Rev. 20, 7–50. 10.1108/eb049150

[B25] Ma J. Ollier-Malaterre A. Lu C.-,q. (2021). The impact of techno-stressors on work–life balance: the moderation of job self-efficacy and the mediation of emotional exhaustion. Comput. Hum. Behav. 122:106811. 10.1016/j.chb.2021.106811

[B26] Meijman T. F. Mulder G. (1998). Psychological aspects of workload, in Handbook of Work and Organizational Psychology, eds Drenth P. J. D. Thierry H. (Hove: Psychology Press), 5–33.

[B27] Nelson D. L. (1990). Individual adjustment to information-driven technologies: a critical review. Manag. Inf. Syst. Q. 14, 79–98. 10.2307/249311

[B28] Podsakoff P. M. MacKenzie S. B. Lee J.-Y. Podsakoff N. P. (2003). Common method biases in behavioral research: a critical review of the literature and recommended remedies. J. Appl. Psychol. 88, 879–903. 10.1037/0021-9010.88.5.879 14516251

[B29] Ragu-Nathan T. S. Tarafdar M. Ragu-Nathan B. S. Qiang T. (2008). The consequences of technostress for end users in organization: conceptual development and empirical validation. Inf. Syst. Res. 19, 417–433. 10.1287/isre.1070.0165

[B30] Richardson K. M. (2017). Managing employee stress and wellness in the new millennium. J. Occup. Health Psychol. 22, 423–428. 10.1037/ocp0000066 28150995

[B31] Sarfraz M. Shah S. G. M. Ivascu L. Qureshi M. A. A. (2020). Explicating the impact of hierarchical CEO succession on small-medium enterprises' performance and cash holdings. Int. J. Finance Econ. 1–15. 10.1002/ijfe.228925855820

[B32] Seligman M. E. P. (2011). Flourish a Visionary New Understanding of Happiness and Well-Being. New York, NY: Simon and Schuster.

[B33] Shah S. G. M. Sarfraz M. Ivascu L. (2021). Assessing the interrelationship corporate environmental responsibility, innovative strategies, cognitive and hierarchical CEO: a stakeholder theory perspective. Corp. Soc. Responsib. Environ. Manag. 28, 457–473. 10.1002/csr.206125855820

[B34] Srivastava S. C. Chandra S. Shirish A. (2015). Technostress creators and job outcomes: theorising the moderating influence of personality traits. Inf. Syst. J. 25, 355–401. 10.1111/isj.12067

[B35] Tarafdar M. Cooper C. L. Stich J. F. (2019). The technostress trifecta – techno eustress, techno distress and design: theoretical directions and an agenda for research. Inf. Syst. J. 29, 6–42. 10.1111/isj.12169

[B36] Tarafdar M. Pullins E. B. Ragu-Nathan T. S. (2014). Examining impacts of technostress on the professional salesperson's behavioural performance. J. Per. Sell. Sales Manag. 34, 51–69. 10.1080/08853134.2013.870184

[B37] Tarafdar M. Tu Q. Ragu-Nathan B. S. Ragu-Nathan T. S. (2007). The impact of technostress on role stress and productivity. J. Manag. Inf. Syst. 24, 301–328. 10.2753/MIS0742-122224010933166856

[B38] Tarafdar M. Tu Q. Ragu-Nathan T. S. (2010). Impact of Technostress on end user satisfaction and performance. J. Manag. Inf. Syst. 27, 303–334. 10.2753/MIS0742-1222270311

[B39] Tarafdar M. Tu Q. Ragu-Nathan T. S. Ragu-Nathan B. S. (2011). Crossing to the dark side: examining creators, outcomes, and inhibitors of technostress. Comm. ACM 54, 113–120. 10.1145/1995376.1995403

[B40] Weil M. Rosen L. (1997). Technostress: Coping With Technology. New York, NY: Wiley.

[B41] Yaverbaum G. J. (1988). Critical factors in the user involvement: an experimental study of users, organizations and tasks. Manag. Inf. Syst. Q. 12, 75–88. 10.2307/248807

[B42] Zhong B. (2020). Social consequences of internet civilization. Comput. Hum. Behav. 107:106308. 10.1016/j.chb.2020.106308

[B43] Zorn T. E. (2002). The emotionality of information and communication technology implementation. J. Comm. Manag. 7, 160–171. 10.1108/1363254031080729634524104

